# Rostro-caudal networks for sound processing in the primate brain

**DOI:** 10.3389/fnins.2022.1076374

**Published:** 2022-12-15

**Authors:** Sophie K. Scott, Kyle Jasmin

**Affiliations:** ^1^Institute of Cognitive Neuroscience, University College London, London, United Kingdom; ^2^Department of Psychology, Royal Holloway, University of London, Egham, United Kingdom

**Keywords:** speech perception, auditory cortex, neuroanatomy, auditory recognition, sensorimotor processing

## Abstract

Sound is processed in primate brains along anatomically and functionally distinct streams: this pattern can be seen in both human and non-human primates. We have previously proposed a general auditory processing framework in which these different perceptual profiles are associated with different computational characteristics. In this paper we consider how recent work supports our framework.

“Hearing is a form of touch. You feel it through your body, and sometimes it almost hits your face”.

— Evelyn Glennie

“Intermittently she caught the gist of his sentences and supplied the rest from her subconscious, as one picks up the striking of a clock in the middle with only the rhythm of the first uncounted strokes lingering in the mind”.

— F. Scott Fitzgerald, Tender is the Night

Auditory processing in primates is neuroanatomically and functionally bifurcated. There are several models of speech and auditory processing in the human brain built around this principle (Alain et al., 2001; Hickok and Poeppel, 2004; Rauschecker and Scott, 2009; Jasmin et al., 2019), which originated in work on non-human primates (NHP). The NHP literature showed that rostral and caudal auditory cortical fields have distinctly different patterns of anatomical connectivity and different functional properties. For example, cells in rostral superior temporal sulcus were shown to be sensitive to the different kinds of non-human primate vocalizations (recognizing “monkey calls”) while those in the caudal fields were sensitive to the spatial location of the vocalizations (Rauschecker and Tian, 2000). These different functions have been described as “what” and “where/how” pathways within the rostral and caudal fields, respectively. Thus it was discovered that, in the visual system, auditory perception entails more than one kind of processing, with more than one functional goal.

This discovery was transformational for functional imaging studies of human speech processing, not just in terms of the neuroanatomical findings, but because it indicated that different speech perception tasks might recruit different elements of the auditory perception network depending on the task. Tasks that required speech recognition networks, such as single word and sentence perception, consistently show recruitment of rostral temporal lobe fields (Mummery et al., 1999; Wise et al., 1999; Scott et al., 2000). By contrast, tasks that required motor engagement—e.g., speaking aloud, reading aloud in synchrony with other people (Jasmin et al., 2016), or when one’s own voice is acoustically altered during speech production (Meekings and Scott, 2021), caudal auditory fields in humans are recruited. There is also a clear role for caudal auditory fields in representing the spatial location of voices (Hunter et al., 2002): all of these findings are consistent with a role for posterior auditory fields in guiding action.

Auditory neuroscience has made strides to move beyond mere description to computational mechanisms. Indeed, there have been significant advances in our understanding of the potential computational properties that underlie the functional differences seen in rostral/caudal auditory fields. In terms of anatomical connectivity, work by Scott et al. (2017) has shown convincingly that rostral and caudal auditory core, belt and parabelt areas receive different inputs from thalamic nuclei, which follows a caudal-rostral distinction: caudal auditory areas receive input mainly from the auditory thalamus and from the somatosensory thalamus (Hackett et al., 2007): moving rostrally, the medial geniculate body (the auditory thalamic input) drops, proportionally, and rostral auditory fields receive proportionally more input from the medial pulvinar, which receives input from the ascending visual pathway. Moving from caudal to rostral fields, the proportion of responses from subnuclei of the medial geniculate body also changes—from a ventral medial geniculate body dominance in caudal and mid-core auditory cortex, to a rough equivalence of inputs from the ventral medial geniculate body and the posterior dorsal medial geniculate body. Given the sheer complexity of the mammalian ascending auditory pathway, an important step in exploring the computational basis of different patterns of auditory processing is going to entail engaging with the nature of the representations of sound in these cortico-thalamic interactions. Some work on the stimulation of brain stem nuclei has suggested that there may even be processing pathways as early as the cochlear nucleus that have critical importance for speech perception (Moore and Shannon, 2009).

Scott et al. (2011) also showed that the caudal core field (A1) shows more detailed temporal response characteristics than the rostral temporal core area (RT): Neurons in caudal A1 respond faster to the onsets of sounds than rostral RT, and they are also accurate at tracking both fast and slow amplitude modulations. This stands in contrast to rostral RT, which responds more slowly to sound onsets and can only track slower amplitude modulations. Recent electrocorticography (ECoG) in humans are consistent with this macaque findings. Across human auditory cortex, regardless of the nature of the auditory stimuli, the neural responses in caudal auditory fields are fast, transient, and linked to the onsets of sounds, while the neural responses in rostral auditory fields are slow and sustained (Hamilton et al., 2018). We argued in 2019 that these findings suggested a critical role for neuronal temporal responses in different kinds of computational processes on incoming sounds. In caudal fields, the responses to sound onsets are fast and temporally accurate, but not sustained, as responses that are critical to the control of action would need to be. By contrast, in rostral fields, the responses to sound onsets are slow and sustained, which potentially reflects hierarchical patterns of perceptual processing that interact with higher order linguistic and predictive processes.

This work has been recently replicated and extended in humans using fMRI. Zulfiqar et al. (2021) modeled fMRI BOLD responses for different temporal and spectral characteristics of the responses to stimuli. They found that caudal belt regions of the auditory cortex showed responses to natural sound stimuli that were fast but not frequency specific, responding to a broad spectral range. In contrast, rostral belt regions showed more specific spectral responses, and slower onset responses. Further support for this comes from another ECoG paper from Hamilton et al. (2021), which reported the shortest onset responses (generally less than 100 ms) in caudal Heschl’s Gyrus (the location of primary auditory cortex in humans) and posterior superior temporal gyrus fields, and longer onset responses (up to 500 ms) in anterior superior temporal gyrus fields and the planum polare.

These findings strongly suggest that, as we hypothesized in 2019, the caudal/posterior “what/how” auditory pathway is underpinned by distinct computational processes from those of the anterior/rostral “what” pathway. Caudal fields (core and non-core) have responses that are generally fast, transient, and not necessarily specifically associated with particular stimulus characteristics: The responses in rostral fields (core and non-core) are generally slow and sustained and can be much more driven by stimulus specific properties. These distinctions are generalities—as can be seen in the Hamilton et al. (2021) paper, there is some overlap of these responses, but the general pattern is clear: Fast transient caudal responses reflect feed forward networks which are critical to the fast sensory guidance of action; slow, sustained responses in rostral fields likely reflect recognition processes which are slower as they require feedback processes from higher order language areas, which can have a profound effect on speech intelligibility (Obleser et al., 2007). This pattern reflects the overall cortical thickness gradient in the temporal lobes, such that primary auditory cortex is thin, with fewer feedback connections that cross cortical layers, whereas moving rostrally the cortex is thicker and has a higher ratio of feedback connections (Wagstyl et al., 2015).

Several studies have now shown that the rostral recognition “what” pathway, seen for intelligibility in speech, is not only seen for speech: music and other identifiable environmental sounds also recruit the anterior temporal lobes in humans. There is compelling evidence that sound recognition is processed by parallel and distinct streams within these anterior fields (Norman-Haignere et al., 2015, 2022; Boebinger et al., 2021). This strongly suggests that while speech may often appear to dominate in these regions, that may be a function of the predominance of studies that focus on speech, and of the well-established speech processing problems that arise due to damage in left middle temporal artery territory. Using non-speech stimuli can show how speech fits within a wider range of auditory stimuli—in a recent ECoG study, song showed greater responses than speech or instrumental music within these fields (Norman-Haignere et al., 2022). However, a computational framework based on the temporal response properties we have described could be applied to a wide range of auditory stimuli—not necessarily specific to speech, as we have discussed (Jasmin et al., 2019). A challenge for further studies will be to determine the degree to which speech, song, instrumental music and other sound sources recruit distinct pathways, and what the computational properties are that may underlie these. This is all the more critical since there is good evidence that when we hear sounds in normal environments, they are rarely in silence, and rostral auditory areas seem to be key for simultaneously representing different sound sources (Evans et al., 2016).

These different auditory perceptual networks also interact with distributed systems throughout the human brain, including both other perceptual networks (including visual, somatosensory systems), and non-perceptual (including linguistic, emotional, musical networks): In many everyday auditory environments one would imagine that both auditory pathways are continually recruited. For example, during conversational speech, we have suggested that the rostral pathway is recruited to process the voice of the other speaker, feeding into language networks that are also engaged in generating a response, while the caudal pathway is recruited to track the features of the other speaker’s voice (e.g., the rate and the rhythm), such that the planned response is aligned with the talkers voice and a smooth turn taking can managed (Scott et al., 2009). Auditory perception requires multiple kinds of perceptual processes, because the brain needs both to track the meaning of our auditory environments and to guide our production of sound into those environments.

## Author contributions

Both authors listed have made a substantial, direct, and intellectual contribution to the work, and approved it for publication.
